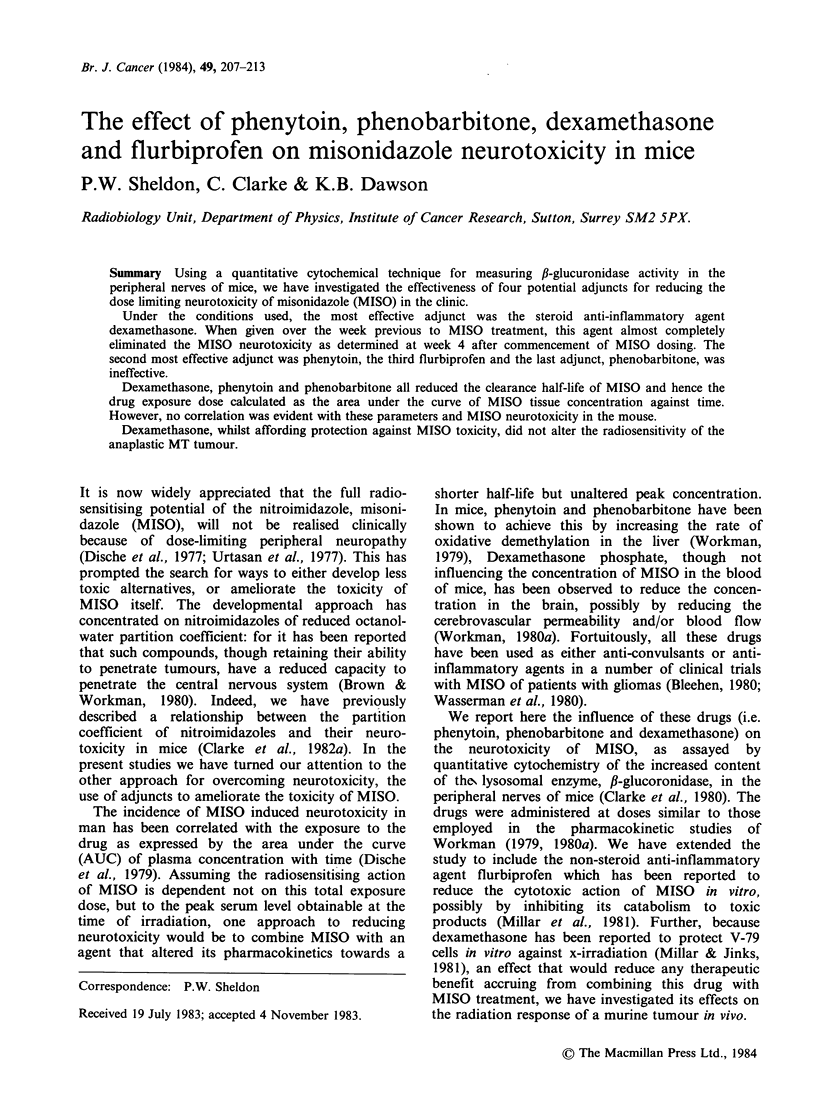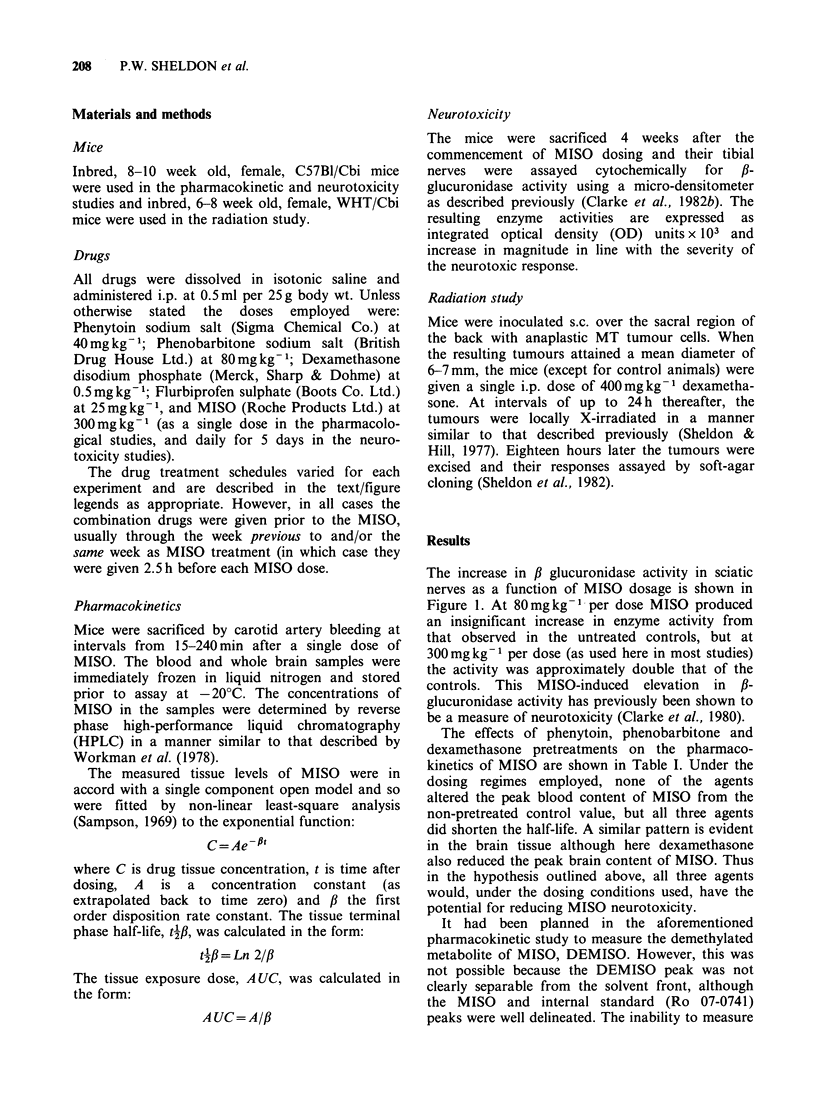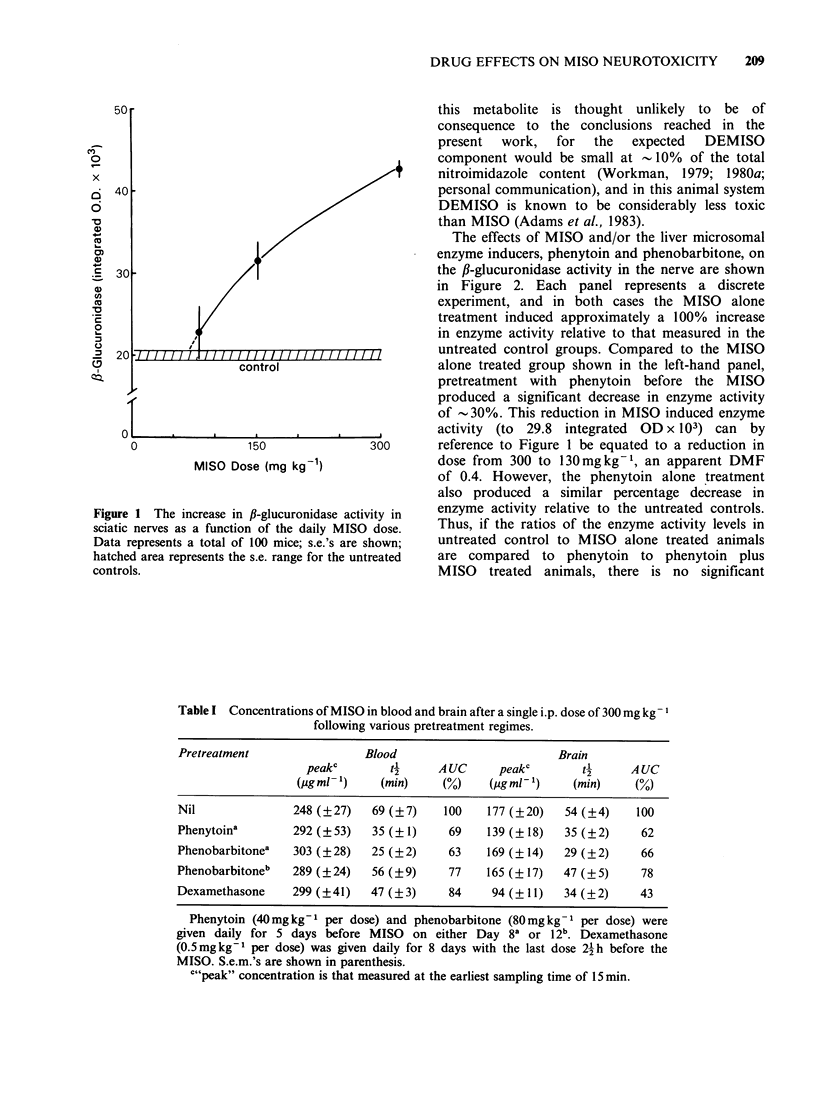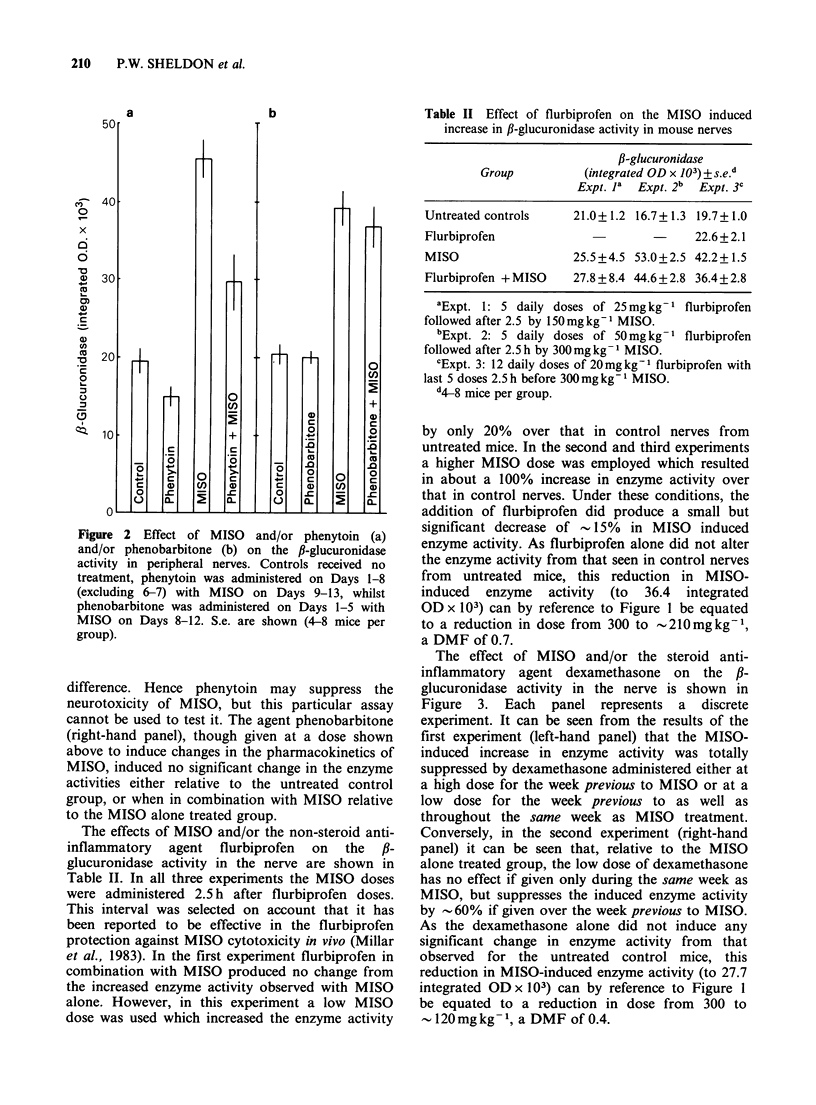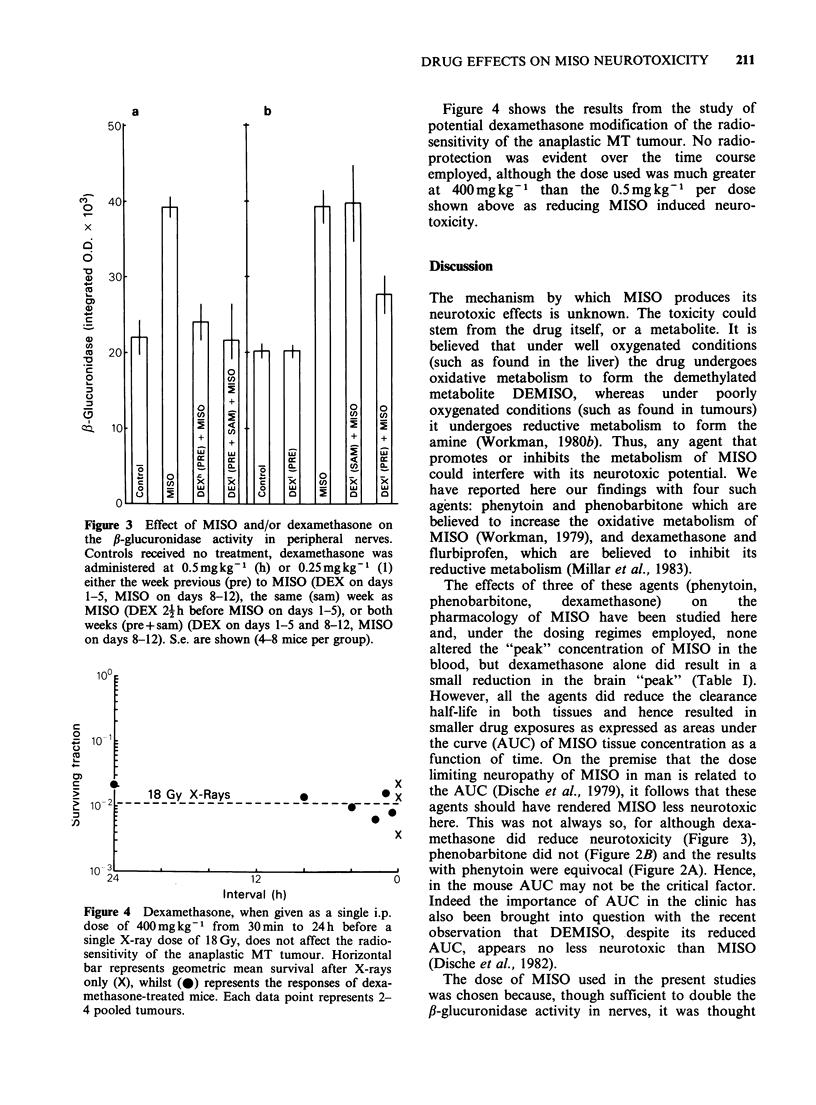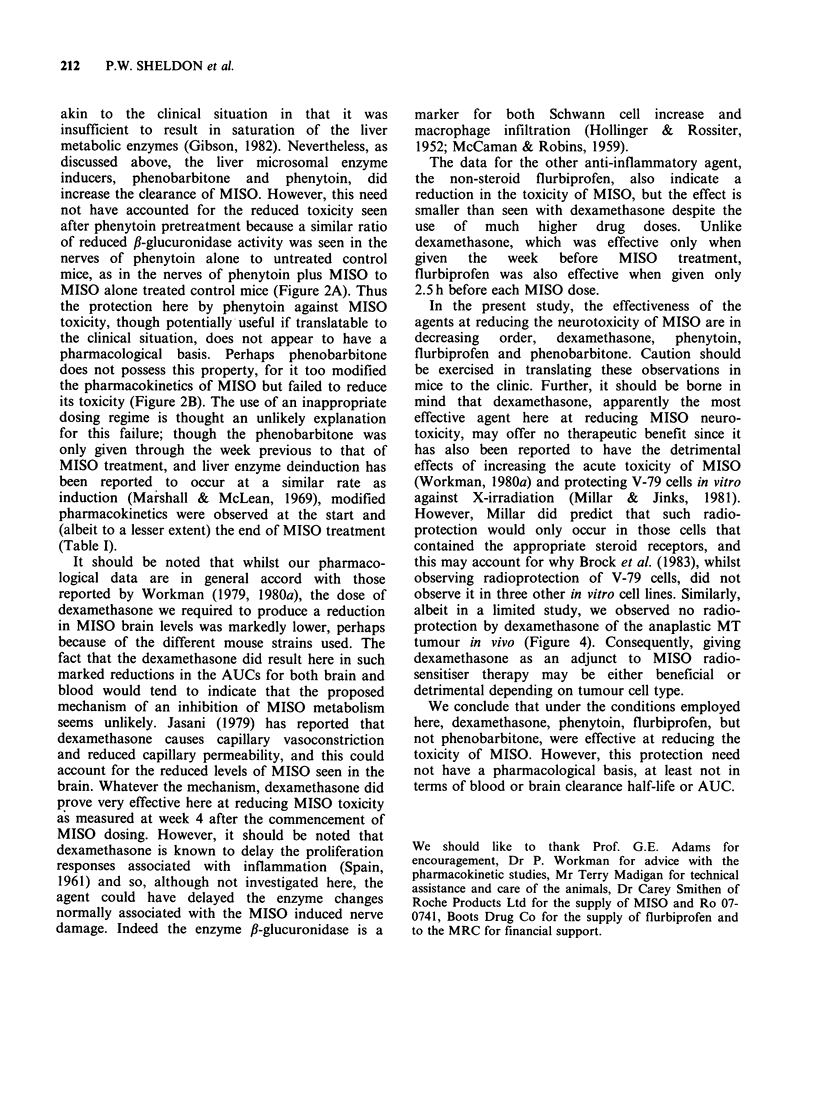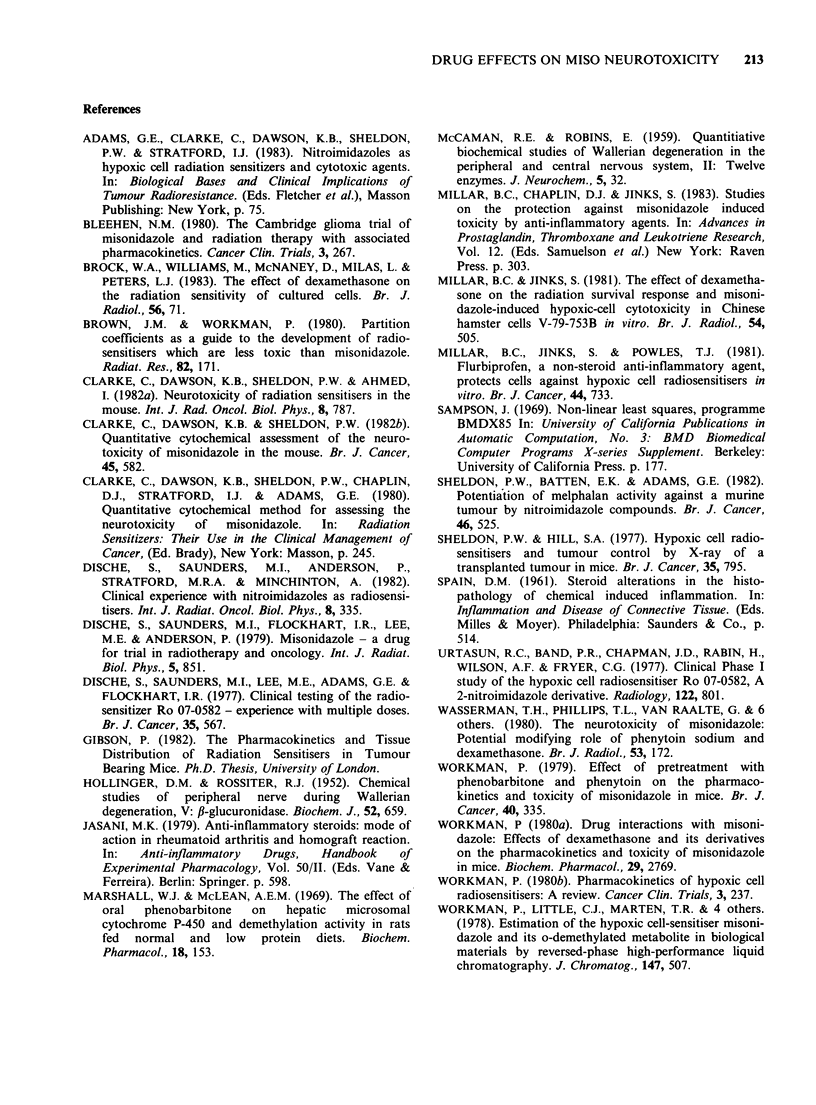# The effect of phenytoin, phenobarbitone, dexamethasone and flurbiprofen on misonidazole neurotoxicity in mice.

**DOI:** 10.1038/bjc.1984.33

**Published:** 1984-02

**Authors:** P. W. Sheldon, C. Clarke, K. B. Dawson

## Abstract

Using a quantitative cytochemical technique for measuring beta-glucuronidase activity in the peripheral nerves of mice, we have investigated the effectiveness of four potential adjuncts for reducing the dose limiting neurotoxicity of misonidazole (MISO) in the clinic. Under the conditions used, the most effective adjunct was the steroid anti-inflammatory agent dexamethasone. When given over the week previous to MISO treatment, this agent almost completely eliminated the MISO neurotoxicity as determined at week 4 after commencement of MISO dosing. The second most effective adjunct was phenytoin, the third flurbiprofen and the last adjunct, phenobarbitone, was ineffective. Dexamethasone, phenytoin and phenobarbitone all reduced the clearance half-life of MISO and hence the drug exposure dose calculated as the area under the curve of MISO tissue concentration against time. However, no correlation was evident with these parameters and MISO neurotoxicity in the mouse. Dexamethasone, whilst affording protection against MISO toxicity, did not alter the radiosensitivity of the anaplastic MT tumour.


*Br. J. Cancer* (1984), **49**, 207–213

### The effect of phenytoin, phenobarbitone, dexamethasone and flurbiprofen on misonidazole neurotoxicity in mice

P.W. Sheldon, C. Clarke & K.B. Dawson

*Radiobiology Unit, Department of Physics, Institute of Cancer Research, Sutton, Surrey SM2 5PX.*

**Summary** Using a quantitative cytochemical technique for measuring β-glucuronidase activity in the peripheral nerves of mice, we have investigated the effectiveness of four potential adjuncts for reducing the dose limiting neurotoxicity of misonidazole (MISO) in the clinic.

Under the conditions used, the most effective adjunct was the steroid anti-inflammatory agent dexamethasone. When given over the week previous to MISO treatment, this agent almost completely eliminated the MISO neurotoxicity as determined at week 4 after commencement of MISO dosing. The second most effective adjunct was phenytoin, the third flurbiprofen and the last adjunct, phenobarbitone, was ineffective.

Dexamethasone, phenytoin and phenobarbitone all reduced the clearance half-life of MISO and hence the drug exposure dose calculated as the area under the curve of MISO tissue concentration against time. However, no correlation was evident with these parameters and MISO neurotoxicity in the mouse.

Dexamethasone, whilst affording protection against MISO toxicity, did not alter the radiosensitivity of the anaplastic MT tumour.

It is now widely appreciated that the full radiosensitising potential of the nitroimidazole, misonidazole (MISO), will not be realised clinically because of dose-limiting peripheral neuropathy (Dische *et al.*, 1977; Urtasan *et al.*, 1977). This has prompted the search for ways to either develop less toxic alternatives, or ameliorate the toxicity of MISO itself. The developmental approach has concentrated on nitroimidazoles of reduced octanol-water partition coefficient: for it has been reported that such compounds, though retaining their ability to penetrate tumours, have a reduced capacity to penetrate the central nervous system (Brown & Workman, 1980). Indeed, we have previously described a relationship between the partition coefficient of nitroimidazoles and their neurotoxicity in mice (Clarke *et al.*, 1982*a*). In the present studies we have turned our attention to the other approach for overcoming neurotoxicity, the use of adjuncts to ameliorate the toxicity of MISO.

The incidence of MISO induced neurotoxicity in man has been correlated with the exposure to the drug as expressed by the area under the curve (AUC) of plasma concentration with time (Dische *et al.*, 1979). Assuming the radiosensitising action of MISO is dependent not on this total exposure dose, but to the peak serum level obtainable at the time of irradiation, one approach to reducing neurotoxicity would be to combine MISO with an agent that altered its pharmacokinetics towards a shorter half-life but unaltered peak concentration. In mice, phenytoin and phenobarbitone have been shown to achieve this by increasing the rate of oxidative demethylation in the liver (Workman, 1979), Dexamethasone phosphate, though not influencing the concentration of MISO in the blood of mice, has been observed to reduce the concentration in the brain, possibly by reducing the cerebrovascular permeability and/or blood flow (Workman, 1980*a*). Fortuitously, all these drugs have been used as either anti-convulsants or anti-inflammatory agents in a number of clinical trials with MISO of patients with gliomas (Bleehen, 1980; Wasserman *et al.*, 1980).

We report here the influence of these drugs (i.e. phenytoin, phenobarbitone and dexamethasone) on the neurotoxicity of MISO, as assayed by quantitative cytochemistry of the increased content of the lysosomal enzyme, β-glucoronidase, in the peripheral nerves of mice (Clarke *et al.*, 1980). The drugs were administered at doses similar to those employed in the pharmacokinetic studies of Workman (1979, 1980*a*). We have extended the study to include the non-steroid anti-inflammatory agent flurbiprofen which has been reported to reduce the cytotoxic action of MISO *in vitro*, possibly by inhibiting its catabolism to toxic products (Millar *et al.*, 1981). Further, because dexamethasone has been reported to protect V-79 cells *in vitro* against x-irradiation (Millar & Jinks, 1981), an effect that would reduce any therapeutic benefit accruing from combining this drug with MISO treatment, we have investigated its effects on the radiation response of a murine tumour *in vivo*.

Correspondence: P.W. Sheldon

Received 19 July 1983; accepted 4 November 1983.

© The Macmillan Press Ltd., 1984

### Materials and methods

### Mice

Inbred, 8–10 week old, female, C57Bl/Cbi mice were used in the pharmacokinetic and neurotoxicity studies and inbred, 6–8 week old, female, WHT/Cbi mice were used in the radiation study.

### Drugs

All drugs were dissolved in isotonic saline and administered i.p. at 0.5 ml per 25 g body wt. Unless otherwise stated the doses employed were: Phenytoin sodium salt (Sigma Chemical Co.) at 40 mg kg$^{-1}$; Phenobarbitone sodium salt (British Drug House Ltd.) at 80 mg kg$^{-1}$; Dexamethasone disodium phosphate (Merck, Sharp & Dohme) at 0.5 mg kg$^{-1}$; Flurbiprofen sulphate (Boots Co. Ltd.) at 25 mg kg$^{-1}$, and MISO (Roche Products Ltd.) at 300 mg kg$^{-1}$ (as a single dose in the pharmacological studies, and daily for 5 days in the neurotoxicity studies).

The drug treatment schedules varied for each experiment and are described in the text/figure legends as appropriate. However, in all cases the combination drugs were given prior to the MISO, usually through the week *previous* to and/or the *same* week as MISO treatment (in which case they were given 2.5 h before each MISO dose.

### Pharmacokinetics

Mice were sacrificed by carotid artery bleeding at intervals from 15–240 min after a single dose of MISO. The blood and whole brain samples were immediately frozen in liquid nitrogen and stored prior to assay at −20°C. The concentrations of MISO in the samples were determined by reverse phase high-performance liquid chromatography (HPLC) in a manner similar to that described by Workman *et al.* (1978).

The measured tissue levels of MISO were in accord with a single component open model and so were fitted by non-linear least-square analysis (Sampson, 1969) to the exponential function:

$$C = Ae^{-\beta t}$$

where $C$ is drug tissue concentration, $t$ is time after dosing, $A$ is a concentration constant (as extrapolated back to time zero) and $\beta$ the first order disposition rate constant. The tissue terminal phase half-life, $t\frac{1}{2}\beta$, was calculated in the form:

$$t\tfrac{1}{2}\beta = Ln\ 2/\beta$$

The tissue exposure dose, $AUC$, was calculated in the form:

$$AUC = A/\beta$$

### Neurotoxicity

The mice were sacrificed 4 weeks after the commencement of MISO dosing and their tibial nerves were assayed cytochemically for $\beta$-glucuronidase activity using a micro-densitometer as described previously (Clarke *et al.*, 1982*b*). The resulting enzyme activities are expressed as integrated optical density (OD) units $\times 10^3$ and increase in magnitude in line with the severity of the neurotoxic response.

### Radiation study

Mice were inoculated s.c. over the sacral region of the back with anaplastic MT tumour cells. When the resulting tumours attained a mean diameter of 6–7 mm, the mice (except for control animals) were given a single i.p. dose of 400 mg kg$^{-1}$ dexamethasone. At intervals of up to 24 h thereafter, the tumours were locally X-irradiated in a manner similar to that described previously (Sheldon & Hill, 1977). Eighteen hours later the tumours were excised and their responses assayed by soft-agar cloning (Sheldon *et al.*, 1982).

### Results

The increase in $\beta$ glucuronidase activity in sciatic nerves as a function of MISO dosage is shown in Figure 1. At 80 mg kg$^{-1}$ per dose MISO produced an insignificant increase in enzyme activity from that observed in the untreated controls, but at 300 mg kg$^{-1}$ per dose (as used here in most studies) the activity was approximately double that of the controls. This MISO-induced elevation in $\beta$-glucuronidase activity has previously been shown to be a measure of neurotoxicity (Clarke *et al.*, 1980).

The effects of phenytoin, phenobarbitone and dexamethasone pretreatments on the pharmacokinetics of MISO are shown in Table I. Under the dosing regimes employed, none of the agents altered the peak blood content of MISO from the non-pretreated control value, but all three agents did shorten the half-life. A similar pattern is evident in the brain tissue although here dexamethasone also reduced the peak brain content of MISO. Thus in the hypothesis outlined above, all three agents would, under the dosing conditions used, have the potential for reducing MISO neurotoxicity.

It had been planned in the aforementioned pharmacokinetic study to measure the demethylated metabolite of MISO, DEMISO. However, this was not possible because the DEMISO peak was not clearly separable from the solvent front, although the MISO and internal standard (Ro 07-0741) peaks were well delineated. The inability to measure

**Figure 1** The increase in β-glucuronidase activity in sciatic nerves as a function of the daily MISO dose. Data represents a total of 100 mice; s.e.'s are shown; hatched area represents the s.e. range for the untreated controls.

this metabolite is thought unlikely to be of consequence to the conclusions reached in the present work, for the expected DEMISO component would be small at ~10% of the total nitroimidazole content (Workman, 1979; 1980*a*; personal communication), and in this animal system DEMISO is known to be considerably less toxic than MISO (Adams *et al.*, 1983).

The effects of MISO and/or the liver microsomal enzyme inducers, phenytoin and phenobarbitone, on the β-glucuronidase activity in the nerve are shown in Figure 2. Each panel represents a discrete experiment, and in both cases the MISO alone treatment induced approximately a 100% increase in enzyme activity relative to that measured in the untreated control groups. Compared to the MISO alone treated group shown in the left-hand panel, pretreatment with phenytoin before the MISO produced a significant decrease in enzyme activity of ~30%. This reduction in MISO induced enzyme activity (to 29.8 integrated $OD \times 10^3$) can by reference to Figure 1 be equated to a reduction in dose from 300 to 130 mg kg$^{-1}$, an apparent DMF of 0.4. However, the phenytoin alone treatment also produced a similar percentage decrease in enzyme activity relative to the untreated controls. Thus, if the ratios of the enzyme activity levels in untreated control to MISO alone treated animals are compared to phenytoin to phenytoin plus MISO treated animals, there is no significant

**Table I** Concentrations of MISO in blood and brain after a single i.p. dose of 300 mg kg$^{-1}$ following various pretreatment regimes.

| *Pretreatment* | *Blood* peak[c] (μg ml$^{-1}$) | *Blood* $t_{\frac{1}{2}}$ (min) | *Blood* AUC (%) | *Brain* peak[c] (μg ml$^{-1}$) | *Brain* $t_{\frac{1}{2}}$ (min) | *Brain* AUC (%) |
|---|---|---|---|---|---|---|
| Nil | 248 (±27) | 69 (±7) | 100 | 177 (±20) | 54 (±4) | 100 |
| Phenytoin[a] | 292 (±53) | 35 (±1) | 69 | 139 (±18) | 35 (±2) | 62 |
| Phenobarbitone[a] | 303 (±28) | 25 (±2) | 63 | 169 (±14) | 29 (±2) | 66 |
| Phenobarbitone[b] | 289 (±24) | 56 (±9) | 77 | 165 (±17) | 47 (±5) | 78 |
| Dexamethasone | 299 (±41) | 47 (±3) | 84 | 94 (±11) | 34 (±2) | 43 |

Phenytoin (40 mg kg$^{-1}$ per dose) and phenobarbitone (80 mg kg$^{-1}$ per dose) were given daily for 5 days before MISO on either Day 8[a] or 12[b]. Dexamethasone (0.5 mg kg$^{-1}$ per dose) was given daily for 8 days with the last dose $2\frac{1}{2}$ h before the MISO. S.e.m.'s are shown in parenthesis.

[c]"peak" concentration is that measured at the earliest sampling time of 15 min.

**Figure 2** Effect of MISO and/or phenytoin (a) and/or phenobarbitone (b) on the β-glucuronidase activity in peripheral nerves. Controls received no treatment, phenytoin was administered on Days 1–8 (excluding 6–7) with MISO on Days 9–13, whilst phenobarbitone was administered on Days 1–5 with MISO on Days 8–12. S.e. are shown (4–8 mice per group).

difference. Hence phenytoin may suppress the neurotoxicity of MISO, but this particular assay cannot be used to test it. The agent phenobarbitone (right-hand panel), though given at a dose shown above to induce changes in the pharmacokinetics of MISO, induced no significant change in the enzyme activities either relative to the untreated control group, or when in combination with MISO relative to the MISO alone treated group.

The effects of MISO and/or the non-steroid anti-inflammatory agent flurbiprofen on the β-glucuronidase activity in the nerve are shown in Table II. In all three experiments the MISO doses were administered 2.5 h after flurbiprofen doses. This interval was selected on account that it has been reported to be effective in the flurbiprofen protection against MISO cytotoxicity *in vivo* (Millar *et al.*, 1983). In the first experiment flurbiprofen in combination with MISO produced no change from the increased enzyme activity observed with MISO alone. However, in this experiment a low MISO dose was used which increased the enzyme activity

**Table II** Effect of flurbiprofen on the MISO induced increase in β-glucuronidase activity in mouse nerves

| Group | *β-glucuronidase (integrated OD × 10³) ± s.e.*[d] Expt. 1[a] | Expt. 2[b] | Expt. 3[c] |
|---|---|---|---|
| Untreated controls | 21.0 ± 1.2 | 16.7 ± 1.3 | 19.7 ± 1.0 |
| Flurbiprofen | — | — | 22.6 ± 2.1 |
| MISO | 25.5 ± 4.5 | 53.0 ± 2.5 | 42.2 ± 1.5 |
| Flurbiprofen + MISO | 27.8 ± 8.4 | 44.6 ± 2.8 | 36.4 ± 2.8 |

[a]Expt. 1: 5 daily doses of 25 mg kg$^{-1}$ flurbiprofen followed after 2.5 by 150 mg kg$^{-1}$ MISO.
[b]Expt. 2: 5 daily doses of 50 mg kg$^{-1}$ flurbiprofen followed after 2.5 h by 300 mg kg$^{-1}$ MISO.
[c]Expt. 3: 12 daily doses of 20 mg kg$^{-1}$ flurbiprofen with last 5 doses 2.5 h before 300 mg kg$^{-1}$ MISO.
[d]4–8 mice per group.

by only 20% over that in control nerves from untreated mice. In the second and third experiments a higher MISO dose was employed which resulted in about a 100% increase in enzyme activity over that in control nerves. Under these conditions, the addition of flurbiprofen did produce a small but significant decrease of ~15% in MISO induced enzyme activity. As flurbiprofen alone did not alter the enzyme activity from that seen in control nerves from untreated mice, this reduction in MISO-induced enzyme activity (to 36.4 integrated OD × 10³) can by reference to Figure 1 be equated to a reduction in dose from 300 to ~210 mg kg$^{-1}$, a DMF of 0.7.

The effect of MISO and/or the steroid anti-inflammatory agent dexamethasone on the β-glucuronidase activity in the nerve is shown in Figure 3. Each panel represents a discrete experiment. It can be seen from the results of the first experiment (left-hand panel) that the MISO-induced increase in enzyme activity was totally suppressed by dexamethasone administered either at a high dose for the week *previous* to MISO or at a low dose for the week *previous* to as well as throughout the *same* week as MISO treatment. Conversely, in the second experiment (right-hand panel) it can be seen that, relative to the MISO alone treated group, the low dose of dexamethasone has no effect if given only during the *same* week as MISO, but suppresses the induced enzyme activity by ~60% if given over the week *previous* to MISO. As the dexamethasone alone did not induce any significant change in enzyme activity from that observed for the untreated control mice, this reduction in MISO-induced enzyme activity (to 27.7 integrated OD × 10³) can by reference to Figure 1 be equated to a reduction in dose from 300 to ~120 mg kg$^{-1}$, a DMF of 0.4.

**Figure 3** Effect of MISO and/or dexamethasone on the β-glucuronidase activity in peripheral nerves. Controls received no treatment, dexamethasone was administered at $0.5 \text{mg kg}^{-1}$ (h) or $0.25 \text{mg kg}^{-1}$ (l) either the week previous (pre) to MISO (DEX on days 1–5, MISO on days 8–12), the same (sam) week as MISO (DEX 2½h before MISO on days 1–5), or both weeks (pre+sam) (DEX on days 1–5 and 8–12, MISO on days 8–12). S.e. are shown (4–8 mice per group).

**Figure 4** Dexamethasone, when given as a single i.p. dose of $400 \text{mg kg}^{-1}$ from 30 min to 24 h before a single X-ray dose of 18 Gy, does not affect the radiosensitivity of the anaplastic MT tumour. Horizontal bar represents geometric mean survival after X-rays only (X), whilst (●) represents the responses of dexamethasone-treated mice. Each data point represents 2–4 pooled tumours.

Figure 4 shows the results from the study of potential dexamethasone modification of the radiosensitivity of the anaplastic MT tumour. No radioprotection was evident over the time course employed, although the dose used was much greater at $400 \text{mg kg}^{-1}$ than the $0.5 \text{mg kg}^{-1}$ per dose shown above as reducing MISO induced neurotoxicity.

### Discussion

The mechanism by which MISO produces its neurotoxic effects is unknown. The toxicity could stem from the drug itself, or a metabolite. It is believed that under well oxygenated conditions (such as found in the liver) the drug undergoes oxidative metabolism to form the demethylated metabolite DEMISO, whereas under poorly oxygenated conditions (such as found in tumours) it undergoes reductive metabolism to form the amine (Workman, 1980*b*). Thus, any agent that promotes or inhibits the metabolism of MISO could interfere with its neurotoxic potential. We have reported here our findings with four such agents: phenytoin and phenobarbitone which are believed to increase the oxidative metabolism of MISO (Workman, 1979), and dexamethasone and flurbiprofen, which are believed to inhibit its reductive metabolism (Millar *et al.*, 1983).

The effects of three of these agents (phenytoin, phenobarbitone, dexamethasone) on the pharmacology of MISO have been studied here and, under the dosing regimes employed, none altered the "peak" concentration of MISO in the blood, but dexamethasone alone did result in a small reduction in the brain "peak" (Table I). However, all the agents did reduce the clearance half-life in both tissues and hence resulted in smaller drug exposures as expressed as areas under the curve (AUC) of MISO tissue concentration as a function of time. On the premise that the dose limiting neuropathy of MISO in man is related to the AUC (Dische *et al.*, 1979), it follows that these agents should have rendered MISO less neurotoxic here. This was not always so, for although dexamethasone did reduce neurotoxicity (Figure 3), phenobarbitone did not (Figure 2*B*) and the results with phenytoin were equivocal (Figure 2A). Hence, in the mouse AUC may not be the critical factor. Indeed the importance of AUC in the clinic has also been brought into question with the recent observation that DEMISO, despite its reduced AUC, appears no less neurotoxic than MISO (Dische *et al.*, 1982).

The dose of MISO used in the present studies was chosen because, though sufficient to double the β-glucuronidase activity in nerves, it was thought akin to the clinical situation in that it was insufficient to result in saturation of the liver metabolic enzymes (Gibson, 1982). Nevertheless, as discussed above, the liver microsomal enzyme inducers, phenobarbitone and phenytoin, did increase the clearance of MISO. However, this need not have accounted for the reduced toxicity seen after phenytoin pretreatment because a similar ratio of reduced β-glucuronidase activity was seen in the nerves of phenytoin alone to untreated control mice, as in the nerves of phenytoin plus MISO to MISO alone treated control mice (Figure 2A). Thus the protection here by phenytoin against MISO toxicity, though potentially useful if translatable to the clinical situation, does not appear to have a pharmacological basis. Perhaps phenobarbitone does not possess this property, for it too modified the pharmacokinetics of MISO but failed to reduce its toxicity (Figure 2B). The use of an inappropriate dosing regime is thought an unlikely explanation for this failure; though the phenobarbitone was only given through the week previous to that of MISO treatment, and liver enzyme deinduction has been reported to occur at a similar rate as induction (Marshall & McLean, 1969), modified pharmacokinetics were observed at the start and (albeit to a lesser extent) the end of MISO treatment (Table I).

It should be noted that whilst our pharmacological data are in general accord with those reported by Workman (1979, 1980*a*), the dose of dexamethasone we required to produce a reduction in MISO brain levels was markedly lower, perhaps because of the different mouse strains used. The fact that the dexamethasone did result here in such marked reductions in the AUCs for both brain and blood would tend to indicate that the proposed mechanism of an inhibition of MISO metabolism seems unlikely. Jasani (1979) has reported that dexamethasone causes capillary vasoconstriction and reduced capillary permeability, and this could account for the reduced levels of MISO seen in the brain. Whatever the mechanism, dexamethasone did prove very effective here at reducing MISO toxicity as measured at week 4 after the commencement of MISO dosing. However, it should be noted that dexamethasone is known to delay the proliferation responses associated with inflammation (Spain, 1961) and so, although not investigated here, the agent could have delayed the enzyme changes normally associated with the MISO induced nerve damage. Indeed the enzyme β-glucuronidase is a marker for both Schwann cell increase and macrophage infiltration (Hollinger & Rossiter, 1952; McCaman & Robins, 1959).

The data for the other anti-inflammatory agent, the non-steroid flurbiprofen, also indicate a reduction in the toxicity of MISO, but the effect is smaller than seen with dexamethasone despite the use of much higher drug doses. Unlike dexamethasone, which was effective only when given the week before MISO treatment, flurbiprofen was also effective when given only 2.5 h before each MISO dose.

In the present study, the effectiveness of the agents at reducing the neurotoxicity of MISO are in decreasing order, dexamethasone, phenytoin, flurbiprofen and phenobarbitone. Caution should be exercised in translating these observations in mice to the clinic. Further, it should be borne in mind that dexamethasone, apparently the most effective agent here at reducing MISO neurotoxicity, may offer no therapeutic benefit since it has also been reported to have the detrimental effects of increasing the acute toxicity of MISO (Workman, 1980*a*) and protecting V-79 cells *in vitro* against X-irradiation (Millar & Jinks, 1981). However, Millar did predict that such radioprotection would only occur in those cells that contained the appropriate steroid receptors, and this may account for why Brock *et al.* (1983), whilst observing radioprotection of V-79 cells, did not observe it in three other *in vitro* cell lines. Similarly, albeit in a limited study, we observed no radioprotection by dexamethasone of the anaplastic MT tumour *in vivo* (Figure 4). Consequently, giving dexamethasone as an adjunct to MISO radiosensitiser therapy may be either beneficial or detrimental depending on tumour cell type.

We conclude that under the conditions employed here, dexamethasone, phenytoin, flurbiprofen, but not phenobarbitone, were effective at reducing the toxicity of MISO. However, this protection need not have a pharmacological basis, at least not in terms of blood or brain clearance half-life or AUC.

We should like to thank Prof. G.E. Adams for encouragement, Dr P. Workman for advice with the pharmacokinetic studies, Mr Terry Madigan for technical assistance and care of the animals, Dr Carey Smithen of Roche Products Ltd for the supply of MISO and Ro 07-0741, Boots Drug Co for the supply of flurbiprofen and to the MRC for financial support.

### References

ADAMS, G.E., CLARKE, C., DAWSON, K.B., SHELDON, P.W. & STRATFORD, I.J. (1983). Nitroimidazoles as hypoxic cell radiation sensitizers and cytotoxic agents. In: *Biological Bases and Clinical Implications of Tumour Radioresistance.* (Eds. Fletcher *et al.*), Masson Publishing: New York, p. 75.

BLEEHEN, N.M. (1980). The Cambridge glioma trial of misonidazole and radiation therapy with associated pharmacokinetics. *Cancer Clin. Trials,* **3,** 267.

BROCK, W.A., WILLIAMS, M., McNANEY, D., MILAS, L. & PETERS, L.J. (1983). The effect of dexamethasone on the radiation sensitivity of cultured cells. *Br. J. Radiol.,* **56,** 71.

BROWN, J.M. & WORKMAN, P. (1980). Partition coefficients as a guide to the development of radiosensitisers which are less toxic than misonidazole. *Radiat. Res.,* **82,** 171.

CLARKE, C., DAWSON, K.B., SHELDON, P.W. & AHMED, I. (1982*a*). Neurotoxicity of radiation sensitisers in the mouse. *Int. J. Rad. Oncol. Biol. Phys.,* **8,** 787.

CLARKE, C., DAWSON, K.B. & SHELDON, P.W. (1982*b*). Quantitative cytochemical assessment of the neurotoxicity of misonidazole in the mouse. *Br. J. Cancer,* **45,** 582.

CLARKE, C., DAWSON, K.B., SHELDON, P.W., CHAPLIN, D.J., STRATFORD, I.J. & ADAMS, G.E. (1980). Quantitative cytochemical method for assessing the neurotoxicity of misonidazole. In: *Radiation Sensitizers: Their Use in the Clinical Management of Cancer,* (Ed. Brady), New York: Masson, p. 245.

DISCHE, S., SAUNDERS, M.I., ANDERSON, P., STRATFORD, M.R.A. & MINCHINTON, A. (1982). Clinical experience with nitroimidazoles as radiosensitisers. *Int. J. Radiat. Oncol. Biol. Phys.,* **8,** 335.

DISCHE, S., SAUNDERS, M.I., FLOCKHART, I.R., LEE, M.E. & ANDERSON, P. (1979). Misonidazole – a drug for trial in radiotherapy and oncology. *Int. J. Radiat. Biol. Phys.,* **5,** 851.

DISCHE, S., SAUNDERS, M.I., LEE, M.E., ADAMS, G.E. & FLOCKHART, I.R. (1977). Clinical testing of the radiosensitizer Ro 07-0582 – experience with multiple doses. *Br. J. Cancer,* **35,** 567.

GIBSON, P. (1982). The Pharmacokinetics and Tissue Distribution of Radiation Sensitisers in Tumour Bearing Mice. *Ph.D. Thesis, University of London.*

HOLLINGER, D.M. & ROSSITER, R.J. (1952). Chemical studies of peripheral nerve during Wallerian degeneration, V: β-glucuronidase. *Biochem. J.,* **52,** 659.

JASANI, M.K. (1979). Anti-inflammatory steroids: mode of action in rheumatoid arthritis and homograft reaction. In: *Anti-inflammatory Drugs, Handbook of Experimental Pharmacology,* Vol. 50/II. (Eds. Vane & Ferreira). Berlin: Springer. p. 598.

MARSHALL, W.J. & McLEAN, A.E.M. (1969). The effect of oral phenobarbitone on hepatic microsomal cytochrome P-450 and demethylation activity in rats fed normal and low protein diets. *Biochem. Pharmacol.,* **18,** 153.

McCAMAN, R.E. & ROBINS, E. (1959). Quantitiative biochemical studies of Wallerian degeneration in the peripheral and central nervous system, II: Twelve enzymes. *J. Neurochem.,* **5,** 32.

MILLAR, B.C., CHAPLIN, D.J. & JINKS, S. (1983). Studies on the protection against misonidazole induced toxicity by anti-inflammatory agents. In: *Advances in Prostaglandin, Thromboxane and Leukotriene Research,* Vol. 12. (Eds. Samuelson *et al.*) New York: Raven Press. p. 303.

MILLAR, B.C. & JINKS, S. (1981). The effect of dexamethasone on the radiation survival response and misonidazole-induced hypoxic-cell cytotoxicity in Chinese hamster cells V-79-753B *in vitro. Br. J. Radiol.,* **54,** 505.

MILLAR, B.C., JINKS, S. & POWLES, T.J. (1981). Flurbiprofen, a non-steroid anti-inflammatory agent, protects cells against hypoxic cell radiosensitisers *in vitro. Br. J. Cancer,* **44,** 733.

SAMPSON, J. (1969). Non-linear least squares, programme BMDX85 In: *University of California Publications in Automatic Computation, No. 3: BMD Biomedical Computer Programs X-series Supplement.* Berkeley: University of California Press. p. 177.

SHELDON, P.W., BATTEN, E.K. & ADAMS, G.E. (1982). Potentiation of melphalan activity against a murine tumour by nitroimidazole compounds. *Br. J. Cancer,* **46,** 525.

SHELDON, P.W. & HILL, S.A. (1977). Hypoxic cell radiosensitisers and tumour control by X-ray of a transplanted tumour in mice. *Br. J. Cancer,* **35,** 795.

SPAIN, D.M. (1961). Steroid alterations in the histopathology of chemical induced inflammation. In: *Inflammation and Disease of Connective Tissue.* (Eds. Milles & Moyer). Philadelphia: Saunders & Co., p. 514.

URTASUN, R.C., BAND, P.R., CHAPMAN, J.D., RABIN, H., WILSON, A.F. & FRYER, C.G. (1977). Clinical Phase I study of the hypoxic cell radiosensitiser Ro 07-0582, A 2-nitroimidazole derivative. *Radiology,* **122,** 801.

WASSERMAN, T.H., PHILLIPS, T.L., VAN RAALTE, G. & 6 others. (1980). The neurotoxicity of misonidazole: Potential modifying role of phenytoin sodium and dexamethasone. *Br. J. Radiol.,* **53,** 172.

WORKMAN, P. (1979). Effect of pretreatment with phenobarbitone and phenytoin on the pharmacokinetics and toxicity of misonidazole in mice. *Br. J. Cancer,* **40,** 335.

WORKMAN, P (1980*a*). Drug interactions with misonidazole: Effects of dexamethasone and its derivatives on the pharmacokinetics and toxicity of misonidazole in mice. *Biochem. Pharmacol.,* **29,** 2769.

WORKMAN, P. (1980*b*). Pharmacokinetics of hypoxic cell radiosensitisers: A review. *Cancer Clin. Trials,* **3,** 237.

WORKMAN, P., LITTLE, C.J., MARTEN, T.R. & 4 others. (1978). Estimation of the hypoxic cell-sensitiser misonidazole and its o-demethylated metabolite in biological materials by reversed-phase high-performance liquid chromatography. *J. Chromatog.,* **147,** 507.